# Adipose-Derived Stem Cells Exosomes Improve Fat Graft Survival by Promoting Prolipogenetic Abilities through Wnt/*β*-Catenin Pathway

**DOI:** 10.1155/2022/5014895

**Published:** 2022-05-06

**Authors:** Kexin Chen, Jiachao Xiong, Sha Xu, Minliang Wu, Chunyu Xue, Minjuan Wu, Chuan Lv, Yuchong Wang

**Affiliations:** ^1^Department of Plastic Surgery, Changhai Hospital, Naval Military Medical University, Shanghai 200433, China; ^2^Department of Plastic Surgery, Shanghai East Hospital, Tongji University School of Medicine, Shanghai 200120, China; ^3^Institute of Translational Medicine, Naval Military Medical University, Shanghai 200433, China; ^4^Department of Histology and Embryology, Naval Military Medical University, Shanghai 200433, China; ^5^School of Life Sciences and Technology, Tongji University, Shanghai 200092, China

## Abstract

Autologous fat grafting has been widely used in plastic surgery in recent years, but the unstable retention of fat graft has always been a key clinical problem. Adipose tissue has poor tolerant to ischemia, so the transplanted adipose tissue needs to rebuild blood supply at an early stage in order to survive stably. Our previous study has found that comparing to human foreskin fibroblast exosome (HFF-Exo), human adipose-derived stem cells exosome (hADSC-Exo) can significantly improve the proliferation of vascular endothelial cells and the angiogenic effect of artificial dermal preconstructed flaps. Therefore, the ability of hADSC-Exo to improve the retention of adipose grafts and its potential regenerative mechanism aroused our strong interest. In this study, we applied hADSC-Exo and HFF-Exo to adipose grafts and explored the potential regeneration mechanism through various means such as bioinformatics, immunofluorescence, immunohistochemistry, and adipogenic differentiation. The results showed that hADSC-Exo can significantly promote grafts angiogenesis and adipogenic differentiation of ADSC to improve the retention of fat grafts and may downregulate the Wnt/*β*-catenin signaling pathway to promote the adipogenic differentiation. In summary, our results provide a theoretical basis for the clinical translation of hADSC-Exo in fat grafting.

## 1. Introduction

Autologous fat grafting is widely used for various treatments in plastic surgery, such as depressed scarring, hemifacial atrophy, and facial rejuvenation, due to its good biocompatibility, low tissue rejection rate, and allergic reaction rate [1, 2]. However, the unstable retention of fat grafts has been a key clinical problem to be overcome. Adipose tissue has poor tolerant to ischemia, liposuction will result in the destruction of their original blood supply, and the transplanted adipose tissue needs to rebuild blood supply at an early stage in order to survive stably [3]. Yoshimura et al. [4] found that grafted fat mostly showed hematologic reconstruction only in the peripheral area, while the central area showed fat necrosis and resorption due to insufficient blood supply and a decrease in grafted tissue volume. Therefore, enhancing hematologic reconstruction in the early stages of fat grafting is one of the key factors to promote graft survival.

Adipose-derived stem cell (ADSC) is a type of stem cell derived from adipose tissue with multidirectional differentiation potential, which is widely used in the field of regenerative medicine in recent years because of its easy extraction and expansion and strong tissue repair activity [5]. Numerous studies [6, 7] have shown that cotransplantation of autologous adipose tissue with ADSC, which are capable of paracrine secretion of various cytokines to promote early vascularization, significantly improves the survival rate of adipose grafts. Interestingly, our previous study [8] found that ADSC can differentiate into vascular endothelial cells and enhance the proliferation and migration of vascular endothelial cells to promote the healing of chronic wounds. However, the tumorigenicity of ADSC and the storage and transport of cellular products are still controversial, and there is a lack of uniform standards for the application of ADSC products [9, 10]. Thus, ADSC does not have the conditions for clinical large-scale application.

Exosomes are a class secreted by cells into the extracellular matrix that can carry a variety of biological components such as proteins, lipids, and noncoding RNAs and can be transported to specific target cells or bound to the cell surface to mediate intercellular communication [11, 12]. ADSC-derived exosome (ADSC-Exo) has been used in ischemic injury diseases and has shown good vascular regenerative effects similar to ADSC, and has the advantages of no risk of tumor formation, high stability, and easy storage and transport, making it more promising for clinical application than ADSC-based cell therapies [13]. In our previous study [14], hADSC-Exo significantly promoted the proliferation of vascular endothelial cells and the angiogenic effect of artificial dermal preconstructed flaps compared to human foreskin fibroblast exosome (HFF-Exo). Therefore, the ability of hADSC-Exo to improve the retention of adipose grafts and its potential regenerative mechanism aroused our strong interest.

We therefore applied hADSC-Exo and HFF-Exo to adipose grafts in this study and found that hADSC-Exo were capable of significant graft vascular regeneration and adipogenic differentiation of ADSC to improve the retention of adipose grafts. Meanwhile, bioinformatics technology was used to explore the potential mechanisms of hADSC-Exo that promote the adipogenic differentiation of stem cells.

## 2. Material and Method

### 2.1. Identification of hADSC

The fourth passage of hADSC was selected for flow cytometry analysis for phenotypic identification of hADSC surface markers. CD90 and CD34 (BD Biosciences, San Jose, CA, USA) were used for flow cytometry analysis. We reported the staining steps previously [8].

### 2.2. Isolation of Exosome

Human adipose-derived stem cells (hADSC) with passages of 3-6 and human foreskin fibroblast (HFF) with passages of 3-6 were cultured for obtaining hADSC-Exo and HFF-Exo. The hADSC and HFF were cultured in low-glucose and high-glucose DMEM (HyClone, Utah, USA), respectively, and supplemented with 10% exosome-free fetal bovine serum (FBS) (Gibco, Grand Island, USA) and incubated with 5% CO_2_ at 37°C. Exosomes were isolated from hADSC and HFF culture medium by differential ultracentrifugation as previously described [15]. In brief, differential ultracentrifugation at 300×*g* and 2000×*g* for 10 min, followed by 10,000×*g* for 30 min, followed by 100,000 × g for 70 min twice were all performed at 4°C. The exosome pellets were finally resuspended in 100 *μ*L of phosphate-buffered saline (PBS) and filtered with 0.22 sterile filter before use.

### 2.3. Verification of Exosome

#### 2.3.1. Transmission Electron Microscopy (TEM)

Exosomes were imaged by TEM to identify their morphology. The exosome sample with a volume of 20 *μ*L was prepared and dropped on a copper mesh for 10 min and stained with 20 *μ*L of 3% sodium phosphotungstate solution for 5 min. Then, copper mesh was dried at room temperature for 30 min. Finally, the sample was detected by TEM.

#### 2.3.2. Nanoparticle Tracking Analysis (NTA)

Particle size distribution of exosomes was identified by NTA (ZetaView, Particle Metrix, Germany). The exosome sample is diluted to the appropriate concentration using PBS and detected on the NTA.

#### 2.3.3. Western Blotting

The western blotting instruction was described previously [8]. In brief, the proteins from each sample were extracted and transferred to a PVDF membrane (Cell Signaling Technology, MA, USA). The membrane was initially blocked in 5% bovine serum albumin for 2 h and then incubated with primary CD63 (1 : 2000, Abcam, Cambridge, MA, UK), CD81 (1 : 1000, Cell Signaling Technology, USA), *α*-tubulin (1 : 1000, Cell Signaling Technology, MA, USA), VEGF-A antibody (1 : 1000, Abcam, Cambridge, MA, UK), and *β*-catenin (1 : 1000, Cell Signaling Technology, USA) at 4°C overnight, followed incubated with the appropriate horseradish peroxidase-conjugated secondary antibodies (1 : 3000, Beyotime, China). An Alpha Imager scanner (Tecan, Thermo Fisher Scientific, USA) was used to visualize the protein expression by chemiluminescence.

### 2.4. Fat Grafting In Vivo

A total of nine nude mice (6 weeks old, male) were purchased from the experimental animal center of Navy Military Medical University (Shanghai, China). All experiments were approved by the guidelines of the Health Sciences Animal Policy and Welfare Committee of Navy Military Medical University.

Transplanted fat from human subcutaneous adipose tissue samples were obtained from the abdominal liposuction of a healthy women in the Changhai Hospital affiliated with the Navy Military Medical University and were obtained with informed consent of the patient. Adipose tissue (0.5 mL) added with hADSC-Exo or HFF-Exo (100 *μ*g) was subcutaneously injected in two recipient sites on the dorsal surface. The transplanted fat was observed at 1, 2, and 3 months postoperatively. The weight and volume of survive fat were assessed.

### 2.5. Immunohistochemistry and Immunofluorescence

Adipose tissues were excised at 1, 2, and 3 months after injection and analyzed by histological staining. The transplanted fat tissues were fixed with 4% polymethylene formaldehyde and embedded with paraffin. Hematoxylin and eosin were used for histological observation of the intrinsic structure of the grafted fat [16]. Transplanted fat angiogenesis and proliferative cells were observed by CD31^+^ (1 : 50, Abcam, UK) and Ki67 (1 : 500, Abcam, UK) immunohistochemical staining, respectively. Differentiated fat cells were observed by PPAR*γ* (1 : 250, Abcam, UK) immunofluorescence staining. The immunohistochemical and immunofluorescence assays were performed as reported previously [8]. Immunohistochemical photomicrographs were obtained under a Pannoramic DESK (3D HISTECH, Hungary) and visualized by Pannoramic scanner, and immunofluorescence photomicrographs were obtained with a Zeiss fluorescence microscope (HLA100, Shanghai, China).

### 2.6. Cell Proliferation Assay

Cell proliferation rates were tested using a Cell Counting Kit-8 (CCK-8) assay (Beyotime, Jiangsu, China) and a 5-ethynyl-2 deoxyuridine (EdU) assay kit (RiboBio, Guangzhou, China). HUVECs (2000 cells/well) were seeded in a 96-well plate and incubated with different concentrations of hADSC-Exo (50, 100 or 200 *μ*g/mL) or equivalent amount of PBS. Cell proliferation was analyzed at 24 h, 48 h, and 72 h after treatment. A 10 *μ*L CCK8 reagent was added to each well, and the cells were incubated for 2 h at each time point. Then, OD value was verified by a microplate reader (Tecan, Thermo Scientific, USA) at 450 nm. The three independent experiments were performed. Based on the CCK8 data, we determined a suitable hADSC-Exo concentration for the follow-up experiment.

An EdU assay kit was performed to compare the proliferative effects hADSC-Exo and HFF-Exo on HUVEC. HUVECs (5 × 10^4^ cells/well) were seeded in a 96-well plate and cultured to reach 70%–80% confluency. Then, HUVECs were cocultured with the suitable hADSC-Exo concentrations and equal concentration of HFF-Exo for 72 h, and an EdU assay was performed according to the manufacturer's instructions [17]. Proliferating HUVECs were stained with green fluorescent, and all cell nuclei were stained with blue fluorescent and observed with the Zeiss fluorescence microscope.

### 2.7. Cell Migration Assay

Transwell assays were performed to compare the migration effects hADSC-Exo and HFF-Exo on HUVEC. HUVECs (5 × 10^4^ cells, 200 *μ*L) resuspended in FBS-free medium and added to the Transwell insert, and the well was added with medium containing 5% exosome-free FBS and hADSC-Exo/HFF-Exo (800 *μ*L). After culture for 18 h, HUVECs were fixed and stained with 0.1% crystalline violet, and the cells that did not cross the membrane were carefully wiped to remove and imaged. The migration cells were measured by using ImageJ software (Bethesda, MD, USA).

### 2.8. Adipogenic Differentiation Assay

The hADSCs (5 × 10^5^ cells) were seeded in 12-well plates and cultured with medium containing 5% exosome-free FBS added with hADSC-Exo or HFF-Exo until their confluency was 70-80%. Then, the cells were cultured with adipogenic differentiation medium (Cyagen, Guangzhou, China) as the manufacturer's instructions for 4 weeks [8]. Then, the cells were fixed and stained with oil red O. The stained cells were washed with PBS to away excess dye and imaged.

### 2.9. Differential Analysis of Gene Expression about Adipogenic Differentiation

The gene expression microarray dataset of hADSC during in vitro adipogenic differentiation (GSE61302), comprised of hADSC samples from 7-day adipogenic differentiation (*n* = 5), 21-day adipogenic differentiation (*n* = 6), and undifferentiated (*n* = 5), was downloaded from the Gene Expression Omnibus (GEO) database (https://www.ncbi.nlm.nih.gov/geo). The dataset was based on the GPL570 platform (Affymetrix Human Genome U133 Plus 2.0 Array), and the mRNA expression data of 21-day adipogenic differentiated hADSC and undifferentiated control were downloaded and used for this study. The differential analysis of gene expression in late-stage adipogenic-differentiated hADSCs compared with undifferentiated cells was performed using the GEO2R analysis tool. An mRNA with a *p* value of <0.05 and a log FC value greater than ±1 was defined as a DEG, and the lists of upregulated and downregulated DEGs were collated for subsequent analysis.

### 2.10. Integration Analysis of Exosomes Contributing to Adipogenic Differentiation

The potential upregulated and downregulated target DEGs were selected, and the protein–protein interaction (PPI) networks were analyzed with the STRING database (https://string- http://db.org/). The hub genes in PPI networks were calculated by using the degree analysis method in the CytoHubba plug-in, and visualized by Cytoscape (version 3.9.0) software [18]. The DAVID database (https://david.ncifcrf.gov/home.jsp) was used to understand the biological function of genes, including gene ontology (GO) and Kyoto Encyclopedia of Genes and Genomes (KEGG) pathway enrichment [19]. GO annotation analysis comprised of molecular function (MF), biological process (BP), and cell component (CC).

### 2.11. Statistical Analysis

Statistical analysis was performed using SPSS 26.0 and described as mean ± SD. Student's *t*-test were used to examine statistical significance, and a *p* value of <0.05 was considered significant.

## 3. Results

### 3.1. Characterization of hADSC and Exosomes

**Figure 1:**
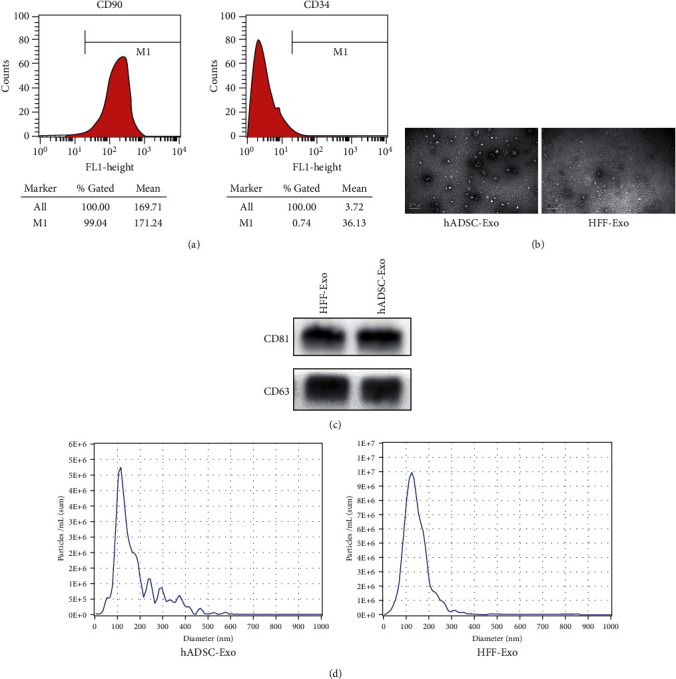
Identification of hADSC, hADSC-Exo, and HFF-Exo. (a) Flow cytometry analysis of cell surface marker CD90 and CD34 was showed in hADSC. (b) The image of exosome was showed through TEM. (c) Western blotting verified the expression of CD63 and CD81 in hADSC-Exo and HFF-Exo. (d) NTA measured the size distributions of hADSC-Exo and HFF-Exo.

To assess the surface marker phenotype of hADSC, the flow cytometry analysis was used to identify the purified hADSC. Approximately 99% of hADSCs were positive for CD90, but negative for CD34 (Figure 1(a)), which suggested that the cultured cells were ADSC. Exosome is a class of membranous vesicles about 30-150 nm in diameter extracted by various methods including ultracentrifugation, exclusion ultrafiltration, density gradient centrifugation, particle size separation, polymer precipitation, and immunoaffinity [15, 20]. In this study, the identification of exosomes was performed by TEM, NTA, and western blotting. TEM confirmed the typical morphology of the exosomes (Figure 1(b)), and NTA showed that the average diameter of hADSC-Exo was 139.5 ± 93.3 nm and HFF-Exo was 131.0 ± 52.2 nm (Figure 1(d)). Moreover, specific membrane protein markers CD63 and CD81 were also highly expressed in both exosomes (Figure 1(c)). These results were all consistent with the characteristics of exosomes.

### 3.2. hADSC-Exo via Proangiogenic and Prolipogenetic Effects Improves Fat Graft Survival In Vivo

**Figure 2:**
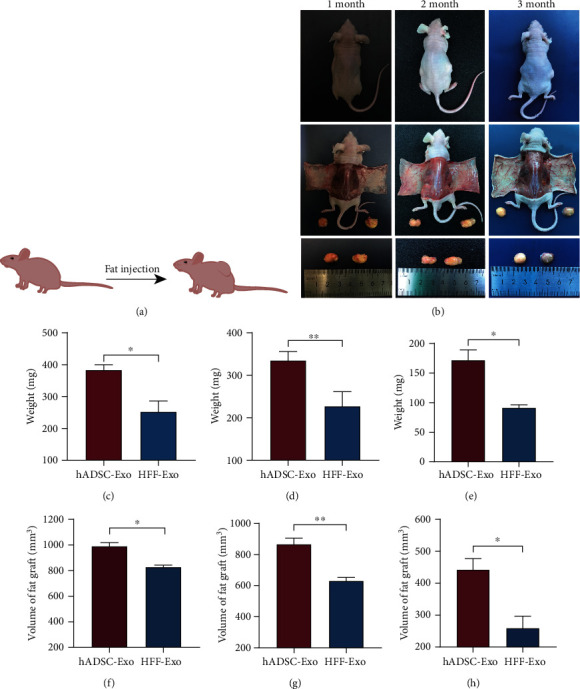
hADSC-Exo enhanced the survival rate of fat grafts in the nude mouse model. (a) The design of the nude mouse model. (b) Gross observation of the fat grafts demonstrated that the hADSC-Exo group (the right side of each picture) had larger graft sizes compared with that in the HFF-Exo group (the left side of each picture). (c–f) Harvesting the fat grafts at 1, 2, and 3 months after fat transplantation, the weight and volume of fat grafts were significantly better in the hADSC-Exo group compared to the HFF-Exo group (∗*p* < 0.05, ∗∗*p* < 0.01, and ∗∗∗*p* < 0.001).

To assess the retention beneficial of hADSC-Exo on adipose grafts, nude mice were used as fat grafting models (Figure 2(a)). The grafts were harvested at 1, 2, and 3 months after fat grafting. A general observation of fat specimens at three time points revealed that the hADSC-Exo had better retention efficiency than HFF-Exo (Figure 2(b)). Then, the weight and volume of grafts were measured and found that the sizes of grafts decreased with the transplantation time, but the hADSC-Exo had better quantitative results than HFF-Exo (Figures 2(c)–2(h)), indicating an excellent effect of hADSC-Exo on grafted fat survival.

**Figure 3:**
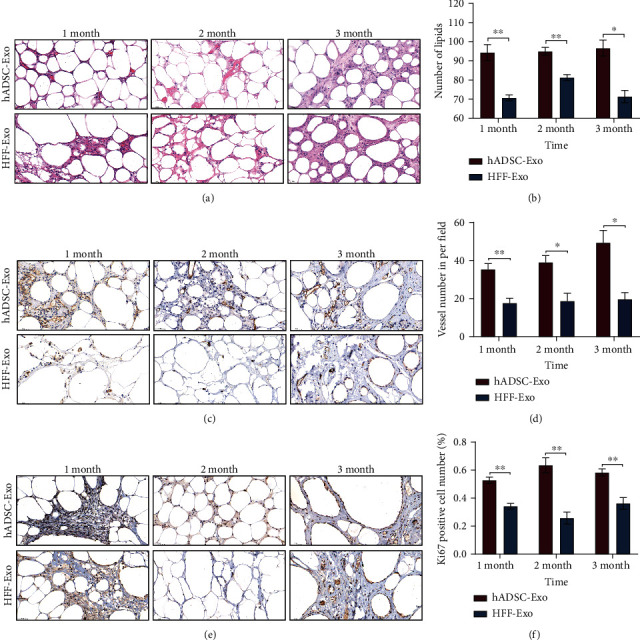
hADSC-Exo promoted the retention and neovascularization of adipose grafts in the nude mice fat grafting model. (a) HE staining revealed that the transplanted fat in the hADSC-Exo groups showed higher adipose number and more complete adipose morphological structure compared to the HFF-Exo group. (b) Semiquantitative scale of integrity of HE staining. (c–f) The number of CD31-positive blood vessels and Ki67-positive proliferating cells in the hADSC-Exo group were significantly higher than that in the HFF-Exo group (∗*p* < 0.05, ∗∗*p* < 0.01, and ∗∗∗*p* < 0.001).

For microscopic assessment of the effect of hADSC-Exo in promoting the retention of adipose grafts, HE staining results (Figures 3(a) and 3(b)) revealed that the transplanted fat from hADSC-Exo groups had higher adipose number and more complete adipose morphological structure compared to the HFF-Exo groups. The degree of vascular regeneration and the number of proliferating cells are also important indicators to evaluate the retention of fat grafts. CD31^+^ and Ki67 immunohistochemistry staining were performed and observed that the number of CD31-positive blood vessels and Ki67-positive proliferating cells in the hADSC-Exo group were significantly higher than that in the HFF-Exo group (Figures 3(c)–3(f)).

### 3.3. Proangiogenic Effect of hADSC-Exo In Vitro

**Figure 4:**
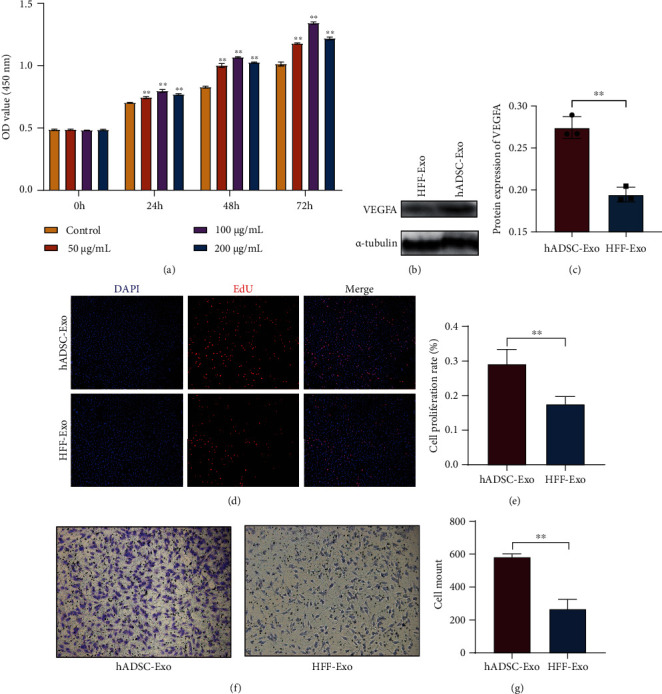
Proangiogenic effects of hADSC-Exo in vitro. (a) The CCK8 revealed that 100 *μ*g/mL of hADSC-Exo had the most significant promotion effect on the proliferation of HUVEC, and this promotion effect persisted with increasing culture time. (b, c) The expression levels of VEGFA genes in fat grafts were treated with hADSC-Exo or HFF-Exo. (d) Proliferating HUVECs were stained with red fluorescence, and all nuclei were stained with blue fluorescence. (e) The cell proliferation rate of hADSC-Exo group was higher than that of HFF-Exo group. (f, g) The migration of HUVEC in the hADSC-Exo group was better than that in the HFF-Exo group. (∗∗*p* < 0.01).

To examine the proangiogenic capacity of hADSC-Exo and HFF-Exo, HUVEC proliferation and migration assays were performed. We first examined the effect of hADSC-Exo on HUVEC proliferation using CCK8 cell proliferation assay to determine the optimal concentration of exosomes for subsequent experiments. The results showed that 100 *μ*g/mL of hADSC-Exo had the most significant promotion effect on HUVEC proliferation, and this promotion effect persisted with increasing culture time (Figure 4(a)). Therefore, we used 100 *μ*g/mL as concentration of hADSC-Exo and HFF-Exo for subsequent experiments. EdU proliferation assays and Transwell assays were used to evaluate the effect of both exosomes on HUVEC proliferation and migration, respectively. Notably, hADSC-Exo were able to promote HUVEC proliferation and migration more effectively than HFF-Exo (Figures 4(d)–4(g)).

VEGFA was initially shown to be an endothelial growth factor and a regulator of vascular permeability [21] and is closely associated with angiogenesis in a variety of conditions, including tumors and wounds [21, 22]. Thus, the protein expression of VEGFA was evaluated and found that hADSC-Exo group exhibited a higher degree of expression (Figures 4(b) and 4(c)). Our finding indicated that hADSC-Exo significantly promoted angiogenesis in adipose grafts.

### 3.4. Identification of Key Target Genes for Lipogenesis by hADSC-Exo

**Figure 5:**
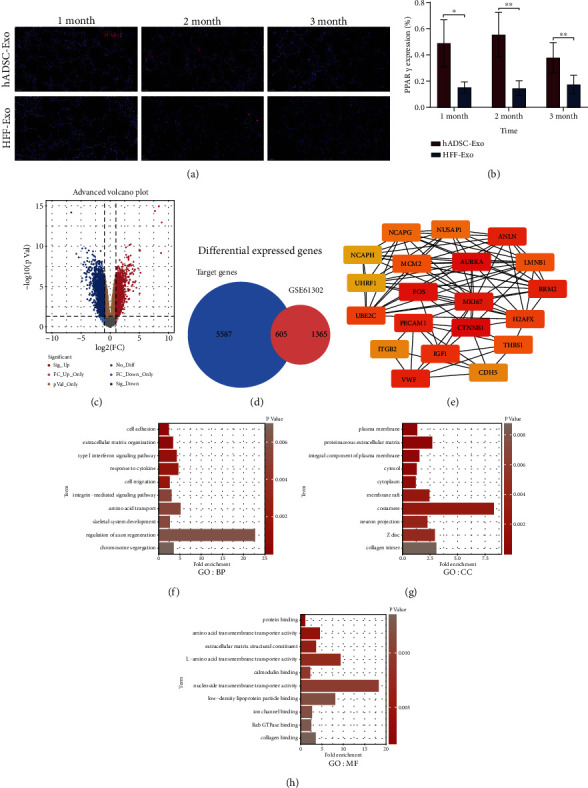
Comprehensive functional analysis of the key DEGs for lipogenesis targeted by hADSC-Exo. (a) The immunofluorescence staining revealed that hADSC-Exo group had evidently significant PPAR*γ* expression compared to the HFF-Exo group. (b) The positive percentage of PPAR*γ* expression in the fat grafts at 1, 2, and 3 months were calculated. (c) Volcano plot of the GSE61302 microarray dataset. (d) Venn diagram of key genes which may target by hADSC-Exo in the process of adipogenic differentiation of ADSC. (e) The mapped network for the top 20 hub genes in protein–protein interaction network. (f) The category of “biological process” of the target DEGs. (g) The category of “cellular component” of the target DEGs. (h) The category of “molecular function” of the target DEGs (∗*p* < 0.05 and ∗∗*p* < 0.01).

After observing that the hADSC-Exo group had superior adipose number and structural integrity, whether hADSC-Exo could promote adipogenic differentiation of the original ADSC in adipose tissue has aroused our strong interest. Therefore, we performed the immunofluorescence staining of PPAR*γ* in fat grafts to observe the status of adipogenic differentiation. The results showed that hADSC-Exo group had evidently significant PPAR*γ* expression compared to the HFF-Exo group (*p* < 0.05) (Figures 5(a) and 5(b)).

To explore the potential mechanisms of lipogenic differentiation of ADSC, the GSE61302 expression profiling data were downloaded from the GEO database, and 1970 DEGs were identified by differential analysis between undifferentiated human primary ADSCs and mature adipocytes (Figures 5(c) and 5(d)). And the top ten upregulated and downregulated DEGs were selected and listed in Tables 1 and 2, respectively. Combined with our previous miRNA sequencing data on hADSC-Exo and HFF-Exo, a total of 605 DEGs may be targeted by hADSC-Exo in the process of adipogenic differentiation of ADSC (Figure 5(d)).

### 3.5. Comprehensive Functional Analysis of the Key Genes Targeted by hADSC-Exo

PPI network is significant for understanding the mechanisms of biosignal and the functional connections between proteins under specific physiological conditions. In this study, the PPI network analysis was performed on the 605 key genes through STRING database. Subsequently, the hub genes with the highest degree of the first 20 nodes were obtained by the degree algorithm (Table 3) and visualized with Cytoscape software (Figure 5(e)). Regarding these hub genes, CTNNB1, AURKA, FOS, MKI67, and VWF were the top five hub genes.

**Figure 6:**
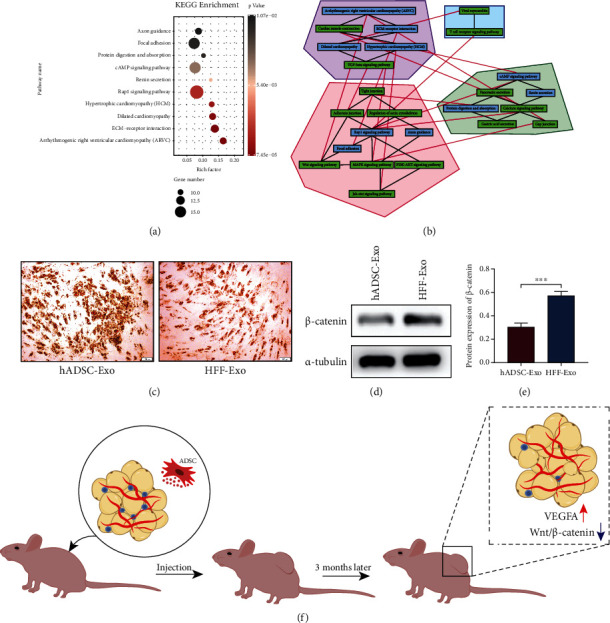
hADSC-Exo promoted adipogenic differentiation of ADSC in adipose grafts. (a) The category of “KEGG enrichment” of the target DEGs. (B) Clusters of pathways each one presented by a different color. The black lines are the connections between nodes that form the identified lusters, while the red ones are those excluded by the algorithm. (c) Cell adipogenic differentiation assays showed that hADSC-Exo significantly promote ADSC adipogenic differentiation. (d, e) The expression levels of *β*-catenin in fat grafts cotransplanted with hADSC-Exo or HFF-Exo. (f) A schematic diagram shows how hADSC-Exo promotes the angiogenesis of fat grafts and maintains the adipogenic differentiation ability of ADSC to promote the retention of fat grafts.

Furthermore, GO annotation and KEGG pathway analysis were performed to further reveal the enrichment status of the DEGs in BP, MF, CC, and KEGG pathways through the DAVID database. With regard to the “biological process” category (Figure 5(f)), it was demonstrated that most of the DEGs were mainly involved in extracellular matrix organization, cell adhesion, and type I interferon signaling pathway. Regarding the CC (Figure 5(g)), the majority of the DEGs were mainly components of plasma membrane, proteinaceous extracellular matrix, integral component of plasma membrane, and costamere. Regarding the MF (Figure 5(h)), the DEGs were mostly involved in amino acid transmembrane transporter activity, extracellular matrix structural constituent, L-amino acid transmembrane transporter activity, and protein binding. Then, the KEGG pathway analysis contributes to study the pathways and functions of the key genes (Figure 6(a)), which mainly were significantly enriched in the signal pathways of ECM-receptor interaction, focal adhesion, and Rap1 signaling pathway.

To further gain our understanding of the pathway interactions under the study, we revealed the direct connections between pathways of interesting and highlight communities (clusters) of pathways through a bioinformatics approach based on graph theory and the edge betweenness clustering algorithm [22–24]. Wnt signaling pathway was identified according to KEGG repository to be highly connected to pathways related to cell–cell adhesion (adherens junction), cell–ECM adhesion (focal adhesion), and the prosurvival pathways of Rap1, MAPK, and PI3K-AKT. (Figure 6(b)).

### 3.6. hADSC-Exo Promote Adipogenic Differentiation of ADSC in Adipose Grafts

Cell differentiation assays were used to further validate the specific effects of hADSC-Exo contributing to adipogenic differentiation and showed that the adipogenic differentiation capacity of ADSCs were preserved and promoted better in the hADSCs-Exo group compared with the HFF-Exo group (Figure 6(c)). The result demonstrated that hADSCs-Exo could maintain and promoted the adipogenic differentiation capacity of ADSCs.

Wnt/*β*-catenin pathway, a key regulatory pathway for adipogenic differentiation, was also detected in adipose grafts. Expression levels of *β*-catenin were significantly lower in the hADSCs-Exo compared with the HFF-Exo group (*p* < 0.05) (Figures 6(d) and 6(e)). The result suggested that hADSC-Exo may regulate the Wnt/*β*-catenin pathway to improve the adipogenesis in fat grafts.

## 4. Discussion

Adipose tissue is particularly sensitive to the ischemic environment, where it is susceptible to necrosis and apoptosis [25]. After fat transplantation, the graft must rebuild its blood supply to survive. Unfortunately, most fat grafts are necrotic, liquefied, and resorbed due to difficulties in reestablishing blood supply [26]. Therefore, early vascular regeneration of fat grafts is promoted as an essential strategy to improve the survival of fat grafts.

Stem cell therapy has high application value in promoting vascularization, but the risk of promoting tumor formation and the difficulty of transformation technology greatly limit its clinical application [27, 28]. Therefore, the search for key active components that can be used directly by replacement stem cells and have angiogenic effects can minimize the above potential problems. Accumulating evidences show that exosomes derived from stem cells have great potential for the treatment of tissue injury, infections, and ischemic diseases [29–31]. Meanwhile, the application of stem cell exosome is free of side effects such as malignancy, risk of vascular occlusion, and immunogenicity, indicating that stem cell exosome-based regenerative therapies are safe and have promising applications [32]. Previous studies have shown that hADSC-Exo repair ischemic tissue disease through provascular regeneration, and our previous work also found that hADSC-Exo can significantly promote the angiogenesis of artificial prefabricated dermis flap [14, 33]. However, whether hADSC-Exo can improve adipose graft survival by promoting adipose graft angiogenesis and its underlying mechanism is not yet clear.

To clarify the effect of hADSC-Exo on adipose graft retention, hADSC-Exo and HFF-Exo were cotransplanted with adipose grafts under the skin of nude mice in this study and observed that hADSC-Exo have a better retention rate than HFF-Exo (Figure 2). Interestingly, we observed that the weight and volume of fat grafts decreased gradually over time in both groups, which could be attributed to the progression of fat grafting, mainly involving cysts, calcification, nodules, fat necrosis, fibrosis, and survival, and eventually stabilization [26, 34, 35]. In addition, fat number and integrity of adipose grafts were significantly higher in the hADSC-Exo group than in the HFF-Exo group (Figures 3(a) and 3(b)). Then, CD31+ and Ki67 immunohistochemistry were used to observe the degree of graft vascularization and the number of proliferating cells and showed that hADSC-Exo group had better vascularization and proliferating cell numbers compared to the HFF-Exo group. Notably, hADSC-Exo was also able to promote the proliferation and migration of HUVEC in vitro, and the protein expression extent of VEGFA was also more significant in adipose grafts in the hADSC-Exo group, which indicates that hADSC-Exo could promote the neovascularization of fat grafts, suggesting that hADSC-Exo could improve retention by promoting graft vascularization.

Adipose tissue is rich in ADSC, which is more resistant to ischemia and hypoxia at the early stage of fat grafting compared with mature adipocytes, vascular endothelial cells, and other and blood-derived cells [36], and is able to regenerate adipose tissue through adipogenesis, angiogenesis, and paracrine effects [37]. In the study, we observed better adipose retention and adipose integrity in the hADSC-Exo group, the ability of hADSC-Exo to promote adipogenic differentiation of ADSC and thus replenish adipose grafts that aroused our interest. Peroxisome proliferator–activated receptor gamma (PPAR*γ*), a major regulator of adipogenesis, plays a dominant role in adipocyte differentiation and acts as a transcription factor expressed in mature adipocytes [38]. Numerous studies have shown that adipose cells apparently cannot form without PPAR*γ* [39–41]. Therefore, we observed the immunofluorescence staining result of PPAR*γ* in two groups of fat grafts and indicated that hADSC-Exo can significantly promote the formation of adipocytes in fat grafts. Indeed, activation of adipose stem cells promotes the survival of fat cells in the regenerated area [26]. However, the potential mechanisms by which hADSC-Exo promote adipogenic differentiation of ADSC are not yet clear.

We hypothesize that hADSC-Exo may target key genes regulating pro-ADSC lipogenic differentiation through its enriched miRNAs. To clarify the potential mechanism, the gene expression microarray datasets of hADSC during in vitro adipogenic differentiation (GSE61302) were downloaded from the GEO database in our study, and 1970 DEGs were identified by differential analysis between undifferentiated human primary ADSCs and mature adipocytes. Meanwhile, our previous study found a total of 43 DE-miRNAs between hADSC-Exo and HFF-Exo. Subsequently, we made an intersection between the target genes predicted by DE-miRNAs and the DEGs to basically identify the key DEGs that may be affected by hADSC-Exo in the process of adipogenic differentiation of ADSC. Then, we explored the biological functions of these genes in adipogenic differentiation through GO annotation and KEGG pathway enrichment analysis. Interestingly, these key genes were directly related to the biological processes of extracellular matrix organization and mainly involved in the signal pathways of ECM-receptor interaction, focal adhesion, and Rap1 signaling pathway. ECM is a multifunctional network, consisting of all kinds of proteins, proteoglycans, and glycosaminoglycans, that regulates stem cell differentiation through reciprocal interactions between cells and components of the ECM [42–44]. The development of preadipocytes to mature adipocytes is accompanied by the remodeling and specific changes of ECM in the process of adipogenic differentiation [45]. Yeung et al. [46] found that the inactivation of Rap1 signaling pathway can promote adipocyte differentiation.

Hub genes as high node degree genes in the PPI network may also play an important role in the development of adipogenic differentiation. Through the analysis of the node degree algorithm in the PPI network, several hub genes with high degree were obtained. Notably, CTNNB1, the largest degree gene in the network, encodes beta-catenin 1 protein and is also a key regulator of Wnt signaling that interacts with E-cadherin and actin cytoskeleton to mediate cell–cell adhesion [47]. Previous studies showed that the activation of Wnt/*β*-catenin signaling can suppress the adipogenic differentiation [48, 49]. In addition, Reggio et al. observed that WNT5a repressed PPAR*γ* expression and adipogenesis through the activation of *β*-catenin signaling [50]. Numerous studies have shown that the activation of PPAR*γ* is associated with a reduction in *β*-catenin levels [51, 52]. Consistently, our study found downregulation of *β*-catenin expression and upregulation of PPAR*γ* in hADSC-Exo cotransplanted adipose grafts. Finally, a graph clustering approach was used to expand the relationship between pathways and confirmed our line of investigation demonstrating that Wnt signaling pathway are highly connected to the pathways involved in cell–cell adhesion (adherens junctions), cell–ECM adhesion (focal adhesion), and are also being connected to the prosurvival pathways of Rap1, MAPK, and PI3K-AKT. Our results provide new insights into the molecular mechanism underlying the role of ADSC-Exo in the survival of fat grafts (Figure 6(f)). However, there were also certain limits in our study. For example, there did not involve the specific miRNA engaged in this pathway in hADSC-Exo; thus, the targeting mechanism is not enough strong. Whereafter, we will use single-cell sequencing technology to compare the cellular and molecular components involved in regeneration in fat grafts, so as to further explore the mechanism of fat graft survival.

## 5. Conclusion

In summary, our findings suggest that hADSC-Exo promotes the angiogenesis of fat grafts and maintains the adipogenic differentiation ability of ADSC to promote the retention of fat grafts and provides a theoretical basis for the clinical translation of hADSC-Exo in fat grafting.

## Figures and Tables

**Table 1:** Top ten upregulated DEGs in GSE61302 between undifferentiated human primary ADSCs and mature adipocytes.

ID	Gene name	Adj. *p* value	*p* value	*t*	*B*	logFC
224354_at	No name	1.45E-09	1.15E-13	3.37E+01	1.91E+01	8.85E+00
234432_at	No name	2.23E-06	7.08E-10	1.65E+01	1.29E+01	8.75E+00
1555623_at	No name	5.86E-11	1.16E-15	4.88E+01	2.11E+01	8.36E+00
1561775_at	No name	1.08E-10	4.30E-15	4.40E+01	2.06E+01	7.69E+00
242625_at	RSAD2	5.69E-05	1.99E-07	1.02E+01	7.65E+00	5.20E+00
205660_at	OASL	1.49E-06	3.54E-10	1.75E+01	1.35E+01	5.15E+00
206336_at	CXCL6	1.79E-03	7.37E-05	5.78E+00	1.74E+00	5.07E+00
210797_s_at	OASL	1.46E-06	2.90E-10	1.78E+01	1.36E+01	5.02E+00
213797_at	RSAD2	2.91E-05	5.71E-08	1.14E+01	8.85E+00	4.82E+00
204533_at	CXCL10	6.79E-04	1.46E-05	6.83E+00	3.37E+00	4.50E+00

**Table 2:** Top ten downregulated DEGs in GSE61302 between undifferentiated human primary ADSCs and mature adipocytes.

ID	Gene name	Adj. *p* value	*p* value	*t*	*B*	logFC
202768_at	FOSB	1.11E-10	6.59E-15	-4.25E+01	2.05E+01	-6.68E+00
203394_s_at	HES1	2.64E-06	8.90E-10	-1.62E+01	1.27E+01	-5.82E+00
206115_at	EGR3	5.00E-06	2.48E-09	-1.49E+01	1.18E+01	-5.41E+00
218839_at	HEY1	2.15E-04	2.22E-06	-8.20E+00	5.27E+00	-4.85E+00
44783_s_at	HEY1	1.34E-04	9.50E-07	-8.86E+00	6.11E+00	-4.82E+00
201693_s_at	EGR1	1.08E-06	1.93E-10	-1.84E+01	1.40E+01	-4.81E+00
209189_at	FOS	1.56E-05	1.91E-08	-1.25E+01	9.89E+00	-4.64E+00
201890_at	RRM2	1.09E-05	9.49E-09	-1.33E+01	1.05E+01	-4.45E+00
216248_s_at	NR4A2	5.83E-05	2.08E-07	-1.02E+01	7.60E+00	-4.44E+00
233180_at	No name	6.39E-05	2.50E-07	-1.00E+01	7.42E+00	-4.24E+00

**Table 3:** Top 20 hub genes in the PPI networks.

Rank	Name	Score
1	CTNNB1	80
2	AURKA	37
3	FOS	36
4	MKI67	33
5	VWF	32
5	PECAM1	32
5	RRM2	32
5	ANLN	32
9	IGF1	30
10	H2AFX	29
10	UBE2C	29
12	THBS1	28
12	LMNB1	28
12	MCM2	28
15	NCAPG	27
15	NUSAP1	27
17	ITGB2	26
17	CDH5	26
19	NCAPH	25
19	UHRF1	25

## Data Availability

The next-generation sequencing data used to support the findings of this study were supplied by Yuchong Wang under license and so cannot be made freely available.
